# Machine Learning to Predict Implant-Based Breast Reconstruction Failure: A Bootstrap-Validated Elastic Net Model

**DOI:** 10.1007/s00266-026-05795-2

**Published:** 2026-04-13

**Authors:** Yanis Berkane, Anna Scarabosio, Glenda G. Caputo, Paul Girard, Roberta Albanese, Damiano Tambasco, Nicolas Bertheuil, Frédéric Bodin, Nicolas Jacquinot, Pier Camilo Parodi

**Affiliations:** 1 Department of Plastic, Reconstructive and Aesthetic Surgery, CHU de Rennes, University of Rennes, 35000 Rennes, France; 2 SITI Laboratory, UMR1236, Etablissement Français du Sang, University of Rennes, 35000 Rennes, France; 3 Plastic and Reconstructive Surgery, Department of Medical Area, University Hospital Santa Maria della Misericordia of Udine, 33100 Udine, Italy; 4 Plastic Surgery Unit, San Carlo di Nancy Hospital, 00165 Rome, Italy; 5 Department of Plastic, Aesthetic and Maxillofacial Surgery, CHRU de Strasbourg, 67091 Strasbourg, France; 6 ICube laboratory, CNRS UMR 7357, MMB, University of Strasbourg, 67091 Strasbourg, France; 7 Department of Gastroenterology, Nancy University Hospital, University of Lorraine, Vandoeuvre-lès-Nancy, F-54052 Nancy, France

**Keywords:** Breast implant, Breast reconstruction, Predictive score, Reconstruction failure, Immediate reconstruction, Radiation, Multivariate analysis

## Abstract

**Background:**

Implant-based reconstruction failure remains a significant complication following breast reconstruction, with substantial implications for patient outcomes. Reliable preoperative risk prediction tools are lacking.

**Methods:**

A retrospective single-center cohort study was conducted, including 381 IBR procedures performed from 2006 to 2023. Reconstruction failure was defined as explantation without immediate replacement within one year. Elastic net regression was used to develop a multivariable model based on age, BMI, smoking status, radiation therapy, chemotherapy, use of expander, and implant plane. Internal bootstrap validation was performed following the 2024 TRIPOD + AI guidelines. Model performance was assessed using AUC, calibration curves, and decision curve analysis. A real-time online calculator was deployed for clinical use.

**Results:**

The mean follow-up was 38.2 ± 27.2 months. The observed IBR failure rate was 5.5% (n = 21). The final model included key predictors such as postoperative radiotherapy, expander use, and an interaction term between age and BMI. Discrimination was strong with an optimism-corrected AUC of 0.856 (95% CI 0.76–0.935). Calibration analysis demonstrated optimal agreement between predicted and observed outcomes, and decision curve analysis confirmed the model’s net clinical benefit over default strategies. The model is accessible via an online tool (https://urls.fr/BIWCeO).

**Conclusions:**

This study presents a robust, internally validated predictive model for implant loss following IBR. The online calculator suggests real-time, individualized risk estimation, supporting preoperative counseling and surgical decision-making in the future. External validation and clinical implementation studies are warranted to confirm its broader applicability.

**Level of Evidence IV:**

This journal requires that authors assign a level of evidence to each article. For a full description of these Evidence-Based Medicine ratings, please refer to the Table of Contents or the online Instructions to Authors www.springer.com/00266.

**Supplementary Information:**

The online version contains supplementary material available at 10.1007/s00266-026-05795-2.

## Introduction

**Fig. 1:**
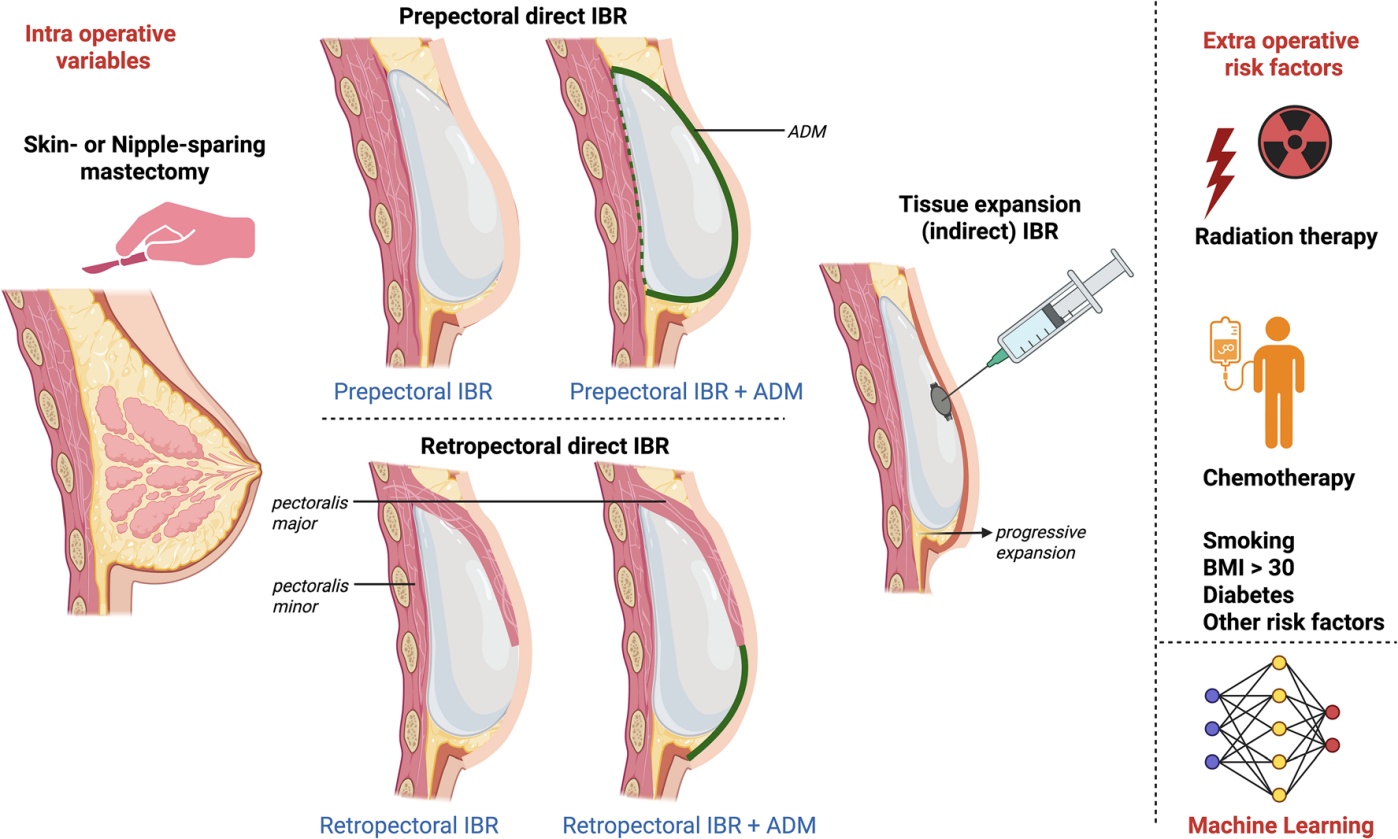
Representative diagram of different implant-based breast reconstruction techniques

Breast reconstruction (BR) represents a crucial step in closing the complex care pathway of patients undergoing mastectomy for breast cancer. While autologous reconstruction has proven better long-term outcomes [1, 2], including patient-reported quality of life [3, 4], the complexity of flap-based procedures and their anatomical requirements lead to implant-based reconstruction (IBR) remaining indispensable in the breast surgeon’s armamentarium [5, 6]. Notably, whether either approach is more suitable in cases with previous or planned radiation therapy is still debatable [7–10]. In IBR, a wide variety of procedures depending on the implant plane, implant type, and the use of supporting materials such as meshes and acellular dermal matrices explain the lack of consensus on the most adequate technique to ensure the best cosmetic outcome while minimizing the risk of postoperative complications [11–13] (Fig. 1). Management of the skin envelope is also a critical variable, with differences in vascular safety between nipple-sparing, skin-sparing, and skin-reducing techniques. More importantly, despite great advances in implant fabrication, surgical techniques, intraoperative protocols, and bacterial infection prophylaxis, major complications with implant exposure and/or infection still occur [14–16]. These consequences often necessitate explantation with delayed reimplantation, resulting in losing the skin envelope and returning to a flat mastectomy [17]. Implant-based reconstruction failure is, therefore, a significant challenge to prevent [2, 18]. Multiple teams have described protocols and controlled studies exploring the outcomes of preventive methods [19–22]. Many of the previously described predictive models have limitations, like the potential for overfitting, poor validation, and/or model building standards [22–27]. To date, no gold-standard predictive model has been developed to help reconstructive surgeons estimate the individual risk of IBR failure and potentially help adapt the surgical technique to each patient. Such tools have been developed in other indications, with real-time scoring allowing simulation of the risk of complications, improving the patient’s education, and correcting significant changeable risk factors before surgery (overweight, smoking status, etc…). Identifying high-risk patients could also lead to specifically reconsidering autologous techniques as safer alternatives.

This work aimed to develop a reliable multivariate predictive model for implant-based reconstruction failure. Variables of interest included age at surgery, body mass index (BMI), smoking status, pre- and planned postoperative radiation therapy and chemotherapy, implant plane, and use of acellular dermal matrices. A large cohort was used, and the multivariable predictive model was created using machine learning-enhanced elastic net regression and bootstrap validation. The cohort study has been reported in line with the STROCSS guidelines [28]. The statistical work was performed following the 2024 TRIPOD + AI guidelines [29, 30]. An online real-time score calculation has been developed to guide IBR decision-making.

## Materials and Methods

### Patients

A single institutional retrospective database was compiled, including all female patients undergoing IBR from June 2006 (reliable electronic medical record system) to March 2023. Ethical approval for this study (protocol N°17487) was provided by the local Ethics Committee. Oral informed consent was obtained for each procedure. Inclusion criteria were patients over 18 years old, undergoing prophylactic or curative, uni- or bilateral, immediate or delayed IBR. Preoperative metrics included height, weight, BMI, smoking status, diabetes, hypertension, preoperative breast radiation, preoperative chemotherapy, indication for postoperative radiotherapy, and indication for postoperative chemotherapy. Intraoperative variables included mastectomy type (nipple-sparing, skin-sparing, skin-reducing), implant type (definitive silicone implant, expander), implant plane [prepectoral, hybrid (submuscular-subcutaneous), and submuscular (full coverage)], use of a synthetic mesh or acellular dermal matrix (Table 1, Supplementary Digital Content), implant or expander immediate volume, and drainage. Finally, postoperative outcomes included implant failure (defined as a reintervention with non-replaced explantation within one year), hematoma, infection, skin necrosis, wound dehiscence, early and late reintervention, capsular contracture, and complementary lipofilling. Patients with incomplete or inaccessible medical records were excluded from the database.

**Table 1:** Descriptive statistics of the included cohort (patient characteristics)

Variable	Mean or n	SD
Age at surg (y)	52.35	12.07
BMI (kg/m2)	22.92	3.5
Active smoking; n (%)	64 (16.89)	–
Preop RxTh; n (%)	43 (11.29)	–
Postop RxTh; n (%)	35 (9.19)	–
Neoadj ChemoTh; n (%)	28 (7.349)	–
Adjuvant ChemoTh; n (%)	158 (41.47)	–
Mastectomy type; n (%)		
Nipple sparing	234 (61.7)	–
Skin sparing	132 (34.7)	–
Skin reducing	13 (3.5)	–
Prophylactic surgery; n (%)	31 (8.1)	–
Implant type; n (%)		
Silicone implant	319 (83.7)	–
Tissue expander	62 (16.3)	–
Use of a matrix; n (%)	246 (64.6)	–
Implant plane; n (%)		
Prepectoral	243 (63.8)	–
Retropectoral	38 (10)	–
Dual plane	100 (26.3)	–
Implant/Expander volume (cc)	322.9	160.52
Follow-up (months)	38.3	27.17
Reconstruction failure; n (%)	21 (5.5)	–

### Data

All patient data were recorded in Excel (v.2024, Microsoft 365, Albuquerque, NM, USA) as categorical variables and were processed using R 4.4.0 (GNU, R Core Team, Vienna, Austria) to create the database matrix. Patients were contacted individually in case of missing data.

### Outcome and Predictors

The predicted outcome was chosen to be reconstruction failure within one year post-surgery. Initial predictors were selected based on the current literature and previous models. Predicting variables included age at surgery, BMI, smoking status, preoperative and postoperative radiotherapy, neoadjuvant and adjuvant chemotherapy, mastectomy type, implant type, matrix use, and implant plane. Assessments were based only on medical records and clinical data, with no blinding applied between predictors and outcomes.

### Sample Size and Missing Data

A sample size of 320 patients included in the training set to develop a new prediction model for the risk of reconstruction failure in plastic surgery was calculated using Richard Riley’s R package. Supplementary Table 2 exposes the sample size calculation technique.

Missing data was managed through discussion between biostatisticians and data managers, achieving secondary data extraction and enabling the model’s training on a complete dataset, enhancing the model’s predictive performance.

### Model Development

**Fig. 2:**
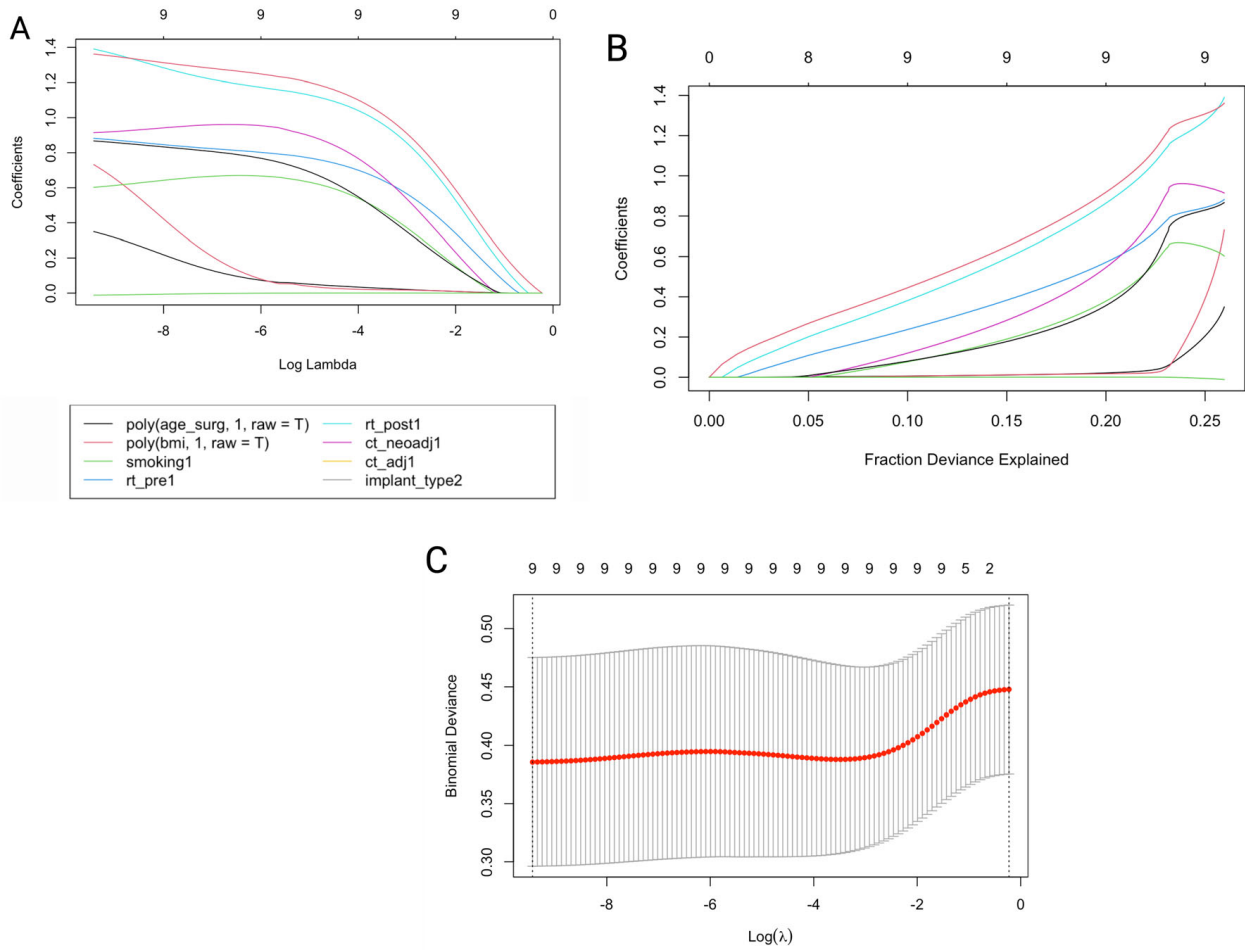
Preliminary model: regularization path curves using elastic net logistic regression

To optimize model evaluation and minimize optimism, we developed an elastic net model based on the entire dataset. This approach, which has been proven superior to stepwise regression [31, 32], enables an optimized balance between relevant variable selection and overfitting prevention [32]. In our model, continuous predictors included age at surgery and BMI, with multiple transformations and interactions evaluated (e.g., polynomial, spline, and fractional polynomial). To prevent overfitting, a simple equation was preferred, excluding higher-order interaction terms and polynomials in favor of a simple linear formula, and the final model’s AUC underwent bootstrap validation. To provide information on the model’s stability and generalizability, preliminary regularization path curves using elastic net regression illustrate how the estimated regression coefficients evolve as the regularization strength (λ) changes (Fig. 2).

### Model Evaluation

According to the recent 2024 TRIPOD + AI reporting guidelines, we proceeded to a model evaluation rather than a model “validation” [29, 30]. Predictive model evaluation aims to estimate the model’s discrimination, calibration, and clinical utility, respectively, with c-statistic, calibration plots, and decision curve analysis. All three metrics were assessed by bootstrap validation from developed candidate models (whose building strategies can be found in Supplementary Table 3) until a final model emerged as the best fit based on its ability to accurately discriminate while maintaining optimal calibration.

Although commonly performed in biomedical research, the Hosmer–Lemeshow test was not used for quantitative calibration assessment, as it does not adequately address overfitting [33]. It was preferred to perform internal validation using bootstrapping to graphically demonstrate calibration between observed real-life probabilities (y-axis) and predicted model-based probabilities (x-axis). Finally, we performed decision curve analysis, enabling a visual demonstration of the clinical utility of predictive models by comparing the current model with two default strategies [34]. Briefly, the comparison was performed over a 0–100% range of decision thresholds to determine if the model outperforms both the “treat none” and “treat all” strategies. Note that the complete mathematical procedure for bootstrapping decision curves has been previously demonstrated with further details on the precise statistical programming code.

## Results

### Surgery and Outcomes

**Fig. 3:**
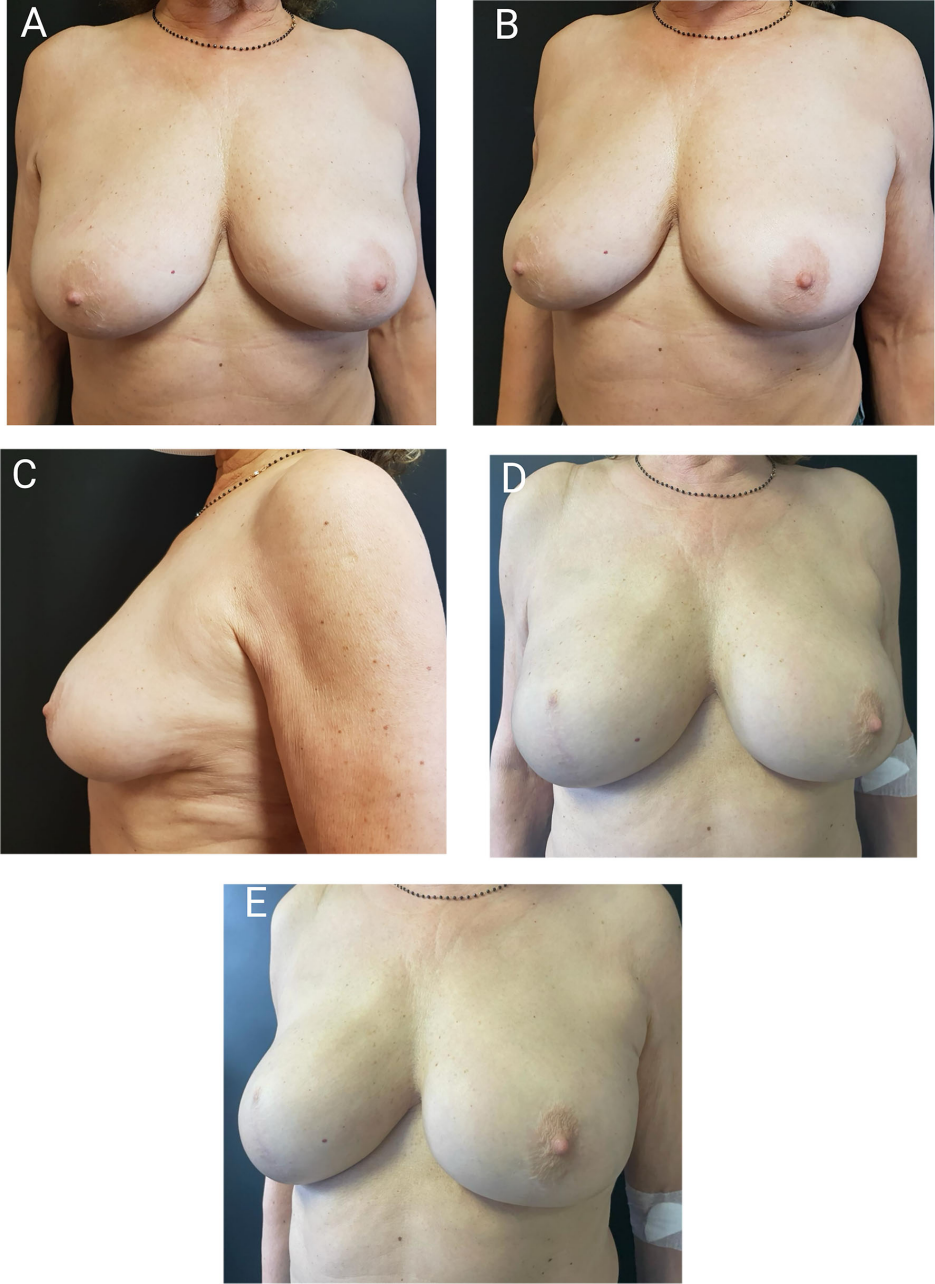
IBR success. Case of a 68-year-old woman diagnosed with a bilateral breast carcinoma. An indication for bilateral mastectomy with a right nipple excision was retained, with no preoperative or adjuvant radiotherapy or chemotherapy. A left nipple-sparing mastectomy and right skin-sparing mastectomy were performed. Immediate reconstruction using prepectoral implants (465 cc, CPG 312, Mentor, Santa Barbara, CA) associated with a ravioli-style Braxon Fast ADM (Braxon, Decomed, Venice, Italy). The patient received adjuvant hormone therapy. No complications or recurrence were recorded at the 24-month follow-up visit, and the patient-reported satisfaction is high

**Fig. 4:**
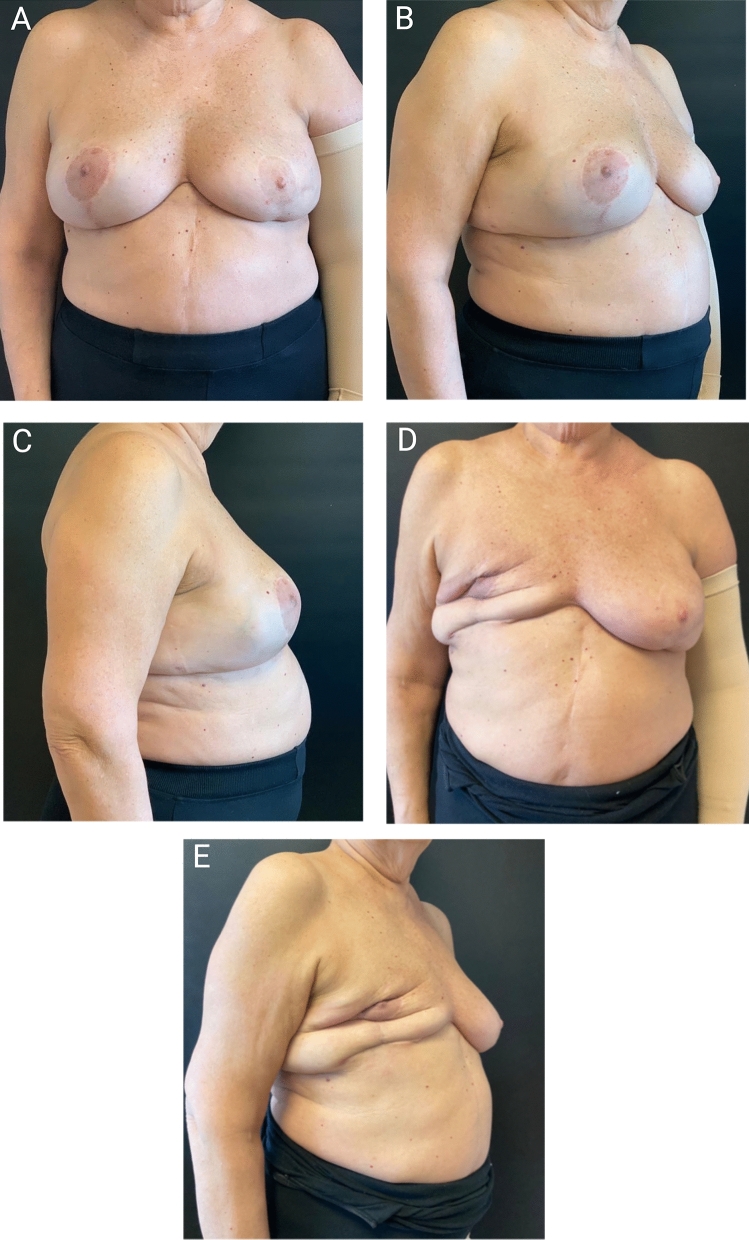
IBR failure. Case of a 74-year-old woman with a history of left conservative treatment with adjuvant radiotherapy for a breast carcinoma of the lower quadrants. She also had received contralateral breast reduction. More than 13 years later, she was diagnosed with a T3N0M0 carcinoma of the right breast, with a retained indication for nipple-sparing mastectomy. It was decided to opt for a delayed reconstruction by placing a tissue expander, due to the redo surgery. Six months later, infection occurred, leading to expander removal and IBR failure. The patient is not interested in an alternative reconstruction

In total, 381 IBR in 353 patients was performed from June 2006 to March 2023 and was included in our data. Figs. 3 and 4 illustrate representative cases of IBR success and failure. The mean follow-up was 38.2 ± 27.2 months. Table 1 displays patient characteristics and outcomes. The mean age at surgery was 52.4 ± 12.1 years, and the mean BMI was 22.9 ± 3.5 kg.m^−2^. Up to 16.8% were active smokers, and 11.3% and 9.3% had neoadjuvant and planned adjuvant radiotherapy, respectively. Neo-adjuvant chemotherapy was wound in 7.3% of cases, while postoperative chemotherapy was planned in 41.5% of them. Concerning mastectomy, 61.4% received nipple-sparing, 34.6% skin-sparing, and 3.4% skin-reduction techniques. Most patients (83.7%) received immediate definitive silicone implants, while 16.3% had expanders placed. The implant plane was prepectoral in 63.8%, submuscular in 26.2%, and hybrid in 10.0% of patients, and a matrix or mesh was used in 35.2% of reconstructions. The mean intraoperative placed volume was 322.9 ± 160.5 ml, and postoperative drainage was pursued for 12.1 ± 4.1 days.

Postoperative outcomes involved 19.4% seroma, including 16.8% minor seroma (no subsequent procedure), while hematoma occurred in 6.3% of cases. Skin necrosis and wound dehiscence were found in 6.8% and 8.1% of included cases, respectively. Infection was diagnosed in 3.7% of patients. In total, reconstruction failure occurred in 21 cases (5.5%), while early reintervention (< 3 months) was necessary in 18.1% versus 26.7% for late reintervention, mainly for cosmetic enhancement.

### Descriptive Statistics

Table 2 depicts the descriptive statistics by outcome (IBR failure) and univariate inferential statistics for variables of interest. Among relevant variables in univariate analysis, age at surgery, pre- and planned postoperative radiotherapy, pre- and postoperative chemotherapy, and the use of a tissue expander showed significant p-values (p<0.05). Missing data following individual patient contact was considered negligible (<3%).

**Table 2:** Descriptive statistics for variables of interest (predictor variables). Univariate inferential statistics were performed for each variable (the outcome was breast reconstruction failure)

Predictor variable	0 (success) *N = 359*	1 (failure) *N = 22*	Combined *N = 381*	Test*
age_surg	_44.9_ 51.8 _59.8_	_48.0_ 56.7 _65.4_	_45.1 _52.0 _60.3_	*F*_1 379_ = 5.7, P=0.017^1^
	52.4 ± 9.8	58.2 ± 10.3	52.7 ± 9.9	
bmi	_20.3_ 22.4 _24.6_	_22.2_ 23.4 _26.5_	_20.3 _22.5 _24.7_	*F*_1 379_ = 3.7, P=0.056^1^
	22.9 ± 3.4	24.4 ± 3.9	23.0 ± 3.5	
smoking	0.16 _57/359_	0.32 _7/22_	0.17 _64/381_	χ^2^_1_ = 3.8, P=0.052^2^
rt_pre	0.10 _36/359_	0.32 _7/22_	0.11 _43/381_	χ^2^_1_ = 9.8, P=0.002^2^
rt_post	0.08 _28/359_	0.32 _7/22_	0.09 _35/381_	χ^2^_1_ = 14, P<0.001^2^
ct_neoadj	0.07 _24/359_	0.18 _4/22_	0.07 _28/381_	χ^2^_1_ = 4, P=0.045^2^
ct_adj	0.40 _144/359_	0.64 _14/22_	0.41 _158/381_	χ^2^_1_ = 4.7, P = 0.03^2^
mastectomy^$^: 1	0.62 _224/359_	0.45 _10/22_	0.61_234/381_	χ^2^_2_ = 2.5, P = 0.28^2^
2	0.34 _122/359_	0.50 _11/22_	0.35 _133/381_	
3	0.04 _13/359_	0.05 _1/22_	0.04 _14/381_	
implant_type^€^2	0.14 _50/359_	0.55 _12/22_	0.16 _62/381_	χ^2^_1_ = 25, P < 0.001^2^
matrix	0.65 _235/359_	0.55 _12/22_	0.65 _247/381_	χ^2^_1_ = 1.1, P = 0.3^2^
implant_plane^£^: 1	0.64 _229/359_	0.64 _14/22_	0.64 _243/381_	χ^2^_2_ = 2.2, P = 0.34^2^
2	0.09 _34/359_	0.18 _4/22_	0.10 _38/381_	
3	0.27 _96/359_	0.18 _4/22_	0.26 _100/381_	

In the age_surg and bmi rows, the pattern (*a* B *c*) represents the lower quartile *a*, the median B, and the upper quartile *c* for continuous variables

*(x ± s)* represents mean ± 1 SD

*Tests used

^1^Wilcoxon test

^2^Pearson test

^$^Mastectomy type (1 = Nipple sparing; 2 = Skin sparing; 3 = Skin reducing)

^€^Implant type (1 = Direct-to-implant; 2 = Expander)

^£^Implant plane (1 = prepectoral; 2 = dual-plane; 3 = submuscular)

### Final Model Specifications and Clinically Ready Equation

Our predictive model for IBR failure was developed using elastic net regularization within a binomial logistic regression framework. Table 3 provides the final specifications, and Eq. 1 demonstrates the final model equation to calculate individual risk using logistic regression.

**Table 3:** Final specifications of the predictive model with predictor variables of interest, corresponding coefficients, and interpretations

Predictor Variable	Coefficient	Interpretation
Intercept	−25.59	Baseline log odds of failure
Age at surgery	0.35	Higher age increases risk
BMI	0.73	Higher BMI increases risk
Smoking	0.60	Active smoking is associated with higher risk
Postoperative radiotherapy	1.39	Large risk increase from postoperative RT
Age-BMI interaction	−0.01	For every 1-unit increase in BMI, the effect of age on the outcome decreases by 0.01 units.

Equation 1. Individual risk score calculation using logistic regression.

1 $$\begin{aligned} {\mathrm{score}} = {\mathrm{logit}}(p) = - 25.59 & + 0.35 \cdot {\mathrm{age\_surg}} \\ & \quad + 0.73 \cdot {\mathrm{bmi}} \\ & \quad + 0.60 \cdot {\mathrm{smoking}} \\ & \quad + 0.88 \cdot {\mathrm{rt\_pre}}1 \\ & \quad + 1.39 \cdot {\mathrm{rt\_post}}1 \\ & \quad + 0.91 \cdot {\mathrm{ct\_neoadji}} \\ & \quad + 0.87 \cdot {\mathrm{ct\_adj}}1 \\ & \quad + 1.36 \cdot {\mathrm{implant\_type}}2 \\ & \quad + 0.01 \cdot {\mathrm{age\_surg}} \cdot {\mathrm{bmi}} \\ \end{aligned}$$

The corresponding predicted probability can be calculated following Eq. 2.

Equation 2. Predicted probability (*p*) of breast reconstruction failure using the individual risk score obtained using Eq. 1.

2 $$p = \frac{1}{{1 + e^{{ - {\mathrm{score}}}} }}$$

The key predictors in this specific model include patient age at surgery, BMI, smoking status, pre- and planned postoperative radiotherapy, neoadjuvant and adjuvant chemotherapy, and implant type. Interaction effects between age and BMI were also considered. The calculated coefficients indicate planned postoperative radiotherapy and implant types as associated with a higher risk of reconstruction failure, while the intercept and age-BMI interaction terms help refine predictions across patient profiles. The intercept (−25.59) represents the baseline log odds of IBR failure when all predictors are null, serving as the starting point for calculating probabilities. The mastectomy design (nipple-sparing, skin-sparing or skin-reducing) was not implemented in the model due to a lack of predictive relevance in this dataset. The BMI, a known higher-order predictor in plastic surgery literature, remained a key variable for predicting reconstruction failure in this model. Additionally, the age-BMI interaction term captures how these two factors jointly influence outcomes. This means that the effect of BMI on reconstruction failure is not constant but varies depending on the patient’s age. Including this interaction allowed the model to provide more accurate and personalized risk predictions across different patient profiles.

The score can be instantly calculated online using the following Uniform Resource Locator: https://urls.fr/BIWCeO and a smartphone-ready QR code (Fig. 1, Supplementary Digital Content). Please note that the variable “Implant Type 2” translates to expander placement.

### Model Performance

**Fig. 5:**
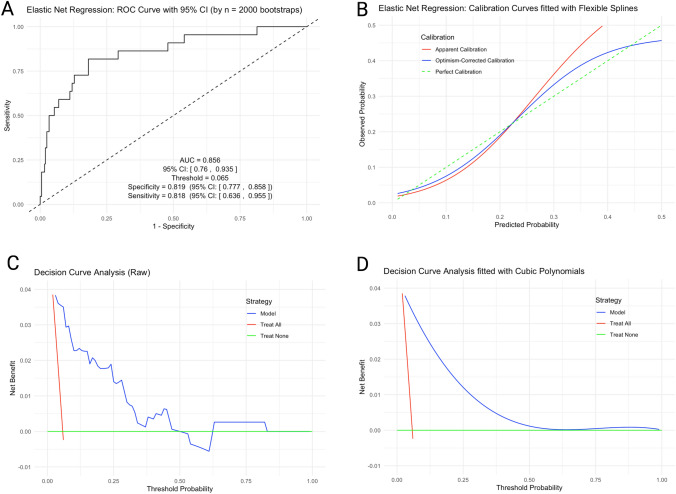
Model performance. The overfitted ROC curve with 95% confidence interval **A** is corrected using bootstrap validation. The curves with flexible splines **B** of the elastic net regression illustrate the model’s calibration. Decision curve analysis **C**, **D** illustrates the clinical pertinence of the model

Figure 5A represents the ROC curve demonstrating optimized model sensitivity (0.818) and specificity (0.819) with a resulting area under the curve (AUC) of 0.856 (95% confidence interval [0.76; 0.935]). It is important to remember the overfitting of the model, imposing its bootstrap validation to take into consideration the optimism-corrected AUC.

### Calibration and Clinical Relevance

Figure 5B depicts the elastic net regression calibration curves, including both apparent and “optimism-corrected” estimations. Clinical relevance through decision-making improvement was evaluated using decision curve analysis. Figures 5C and 5D illustrate the raw and fitted (with cubic polynomials) decision curves, demonstrating that the predictive model for IBR failure outperforms both the “treat all” and “treat none” strategies across a range of clinically relevant threshold probabilities, suggesting clinical relevance by high-risk patient identification, particularly at low threshold probabilities.

## Discussion

Despite the rise of conservative treatments, mastectomy remains the main treatment for advanced breast cancer. While immediate or delayed reconstruction is proposed for most patients, immediate BR might provide better cosmetic results and psychological impact [35–37]. However, the proportion of women undergoing reconstruction following mastectomy remains low: The latest data in the US report 40% reconstruction rates [38], slightly higher than in France (30%) [39]. A lack of information and updates on all available techniques seems to be a major explanation. Breast surgeons must be able to perform or team up with other peers to propose all current BR techniques. Depending on the surgeon’s background, implant-based BR (IBR) could be over-proposed to patients eligible for autologous techniques [24]. This highlights a crucial need for wider multidisciplinary networks to offer a comprehensive treatment choice adapted to each patient.

Implant-based techniques remain the preferred choice in most cases, due to their favorable risk-benefit balance, highlighting the simplicity of the procedure and the mid-term cosmetic and functional outcomes. Unlike implant-based techniques, autologous flaps are not subject to long-term complications such as capsular contracture [40]. Patient-reported outcome measures (PROMs) consistently favor autologous reconstruction in terms of quality of life and body image [41]. However, the prolonged operative times and the need for microsurgical expertise significantly decrease its availability. As highlighted by van Bommel et al. [24], surgeons with limited experience in autologous reconstruction are more likely to recommend implant-based approaches, even in patients who may be better candidates for autologous techniques. This underscores the importance of providing the most appropriate and individualized treatment option. In terms of medico-economic considerations, the long-term costs are comparable between implant-based and autologous reconstruction [42] due to the higher postoperative care requirements and repeated procedures associated with flap-based and fat transfer techniques. Despite lower initial costs, implant-based reconstruction often entails higher postoperative care needs, revisions, and complications over time [42, 43]. There is a clear need for high-quality research, particularly regarding the optimal timing of immediate and autologous reconstruction in relation to chemotherapy and radiotherapy, as well as comparative studies on implant materials and the anatomical planes used for implant placement [44].

In this study, we developed and internally validated a predictive score for IBR failure, incorporating clinical and demographic variables readily available at the preoperative visit. We aimed to provide a practical, data-driven, statistical learning-enhanced tool to support preoperative decision-making and patient counseling in reconstructive breast surgery. The need for such a tool is underscored by the ongoing variability in reconstructive decision-making, which is still strongly influenced by the surgeon’s background and institutional resources [41]. We based our predictive model on a large cohort of 381 implants using various IBR techniques, with a prolonged mean follow-up of 38.2 months. Our cohort’s observed IBR failure rate of 5.5% is consistent with the existing literature, where failure rates typically range between 4 and 10% depending on patient characteristics and surgical technique [14, 45–47]. We based the model by identifying several well-known risk factors from pre-existing evidence, including pre-/postoperative radio- and/or chemotherapy, skin flap and nipple management, expander use, elevated BMI, smoking status, and age as independently associated with reconstruction failure [40, 47–50]. Notably, the age-BMI interaction term was retained in the final model, capturing the compounded effect of these variables, which are often interdependent but insufficiently modeled in previous studies. This model demonstrated strong discriminative performance with an optimism-corrected AUC of 0.856 and reliable calibration, supporting its utility in personalized surgical planning. Unlike earlier attempts at conventional risk calculation [51, 52] and predictive modeling in breast reconstruction [53–55], our work adheres to the 2024 TRIPOD + AI guidelines. It integrates a comprehensive evaluation strategy including bootstrap validation, calibration curves, and decision curve analysis. Besides optimizing the model’s performance, these methods allow for a well-nuanced appraisal of its real-world applicability, especially in guiding indications under clinical uncertainty [31, 56]. This statistical learning method has proven robustness in several fields [57–59] and emerges as a potential gold standard for predictive models [60]. Furthermore, we provide a user-friendly, real-time risk calculator accessible online, allowing peers to integrate objective risk stratification into shared decision-making with patients. A real-time benefit-risk balance can be estimated, leading to preferring IBR in case of a favorable estimated risk, or to bring robust arguments in favor of autologous reconstruction in patients who were initially reluctant.

From a clinical perspective, our findings reinforce the need for nuanced patient selection in implant-based reconstruction, particularly in the setting of planned radiotherapy. While IBR remains a cornerstone of post-mastectomy reconstruction, its complications may compromise esthetic outcomes and patient satisfaction [61, 62]. The outcome criterion (reconstruction failure) was chosen for its strength and potential in orienting decision-making. Other short- and long-term complications, such as hematoma, infection, capsular contracture, or implant displacement, are significant but can be compensated for or treated. In contrast, failure imposes delayed reconstruction, with demonstrated evidence for decreased cosmetic and psychological outcomes [35, 37, 63]. Therefore, this score helps identify high-risk patients preoperatively and provides fair and quantified information that could help redirect them toward autologous options or alternative strategies, including delayed reconstruction or staged approaches. It contributes to shared decision-making and a more personalized approach to breast reconstruction, in line with current calls for higher-quality evidence to guide surgical choices.

As highlighted by Sullivan et al., most of the literature regarding surgical breast reconstruction still suffers from low or moderate strength of evidence, especially concerning the timing of reconstruction relative to adjuvant therapies, implant materials, and implant placement planes [44]. Recent efforts from Yara et al. [64] led to developing a risk prediction model based on a large national database cohort; they only included four risk factors (BMI, smoking status, preoperative radiotherapy, and implant plane). Moreover, despite the power of their cohort, the lack of major postoperative variables such as adjuvant radio- or chemotherapy remains a major limitation. In addition, the calibration results demonstrate good but suboptimal results, and the split-sample internal validation used by the authors has its own limitations. Alternatively, Juyoung et al. used a nomogram-based approach for predicting various postoperative complications following prepectoral direct-to-implant IBR [65]. They based their model using Lasso and Firth-corrected logistic regression, with internal random train-test split validation. Their model was limited only to prepectoral IBR and lacked key performance assessments such as calibration curves and decision curve analysis. In contrast, our study employed elastic net regularization and bootstrap validation, which is substantially more robust than both cited approaches. Our study adhered to the TRIPOD + AI 2024 guidelines, ensuring stronger discrimination, robust internal validation, and broader clinical applicability across all implant-based reconstruction techniques. Additionally, the integration of an online calculator enhances its usability in real-time clinical settings, offering an evidence-based framework to anticipate reconstructive failure and guide the indication for IBR. Its strengths lie not only in the ability to identify high-risk patients but also in modifiable risk factors that may be addressed before surgery, when the timing allows it. Factors such as smoking status, BMI, and timing to radiotherapy or chemotherapy represent actionable elements that, if optimized preoperatively, could significantly reduce the likelihood of reconstructive failure. This score may thus serve not only as a risk stratification tool but also as a lever for implementing targeted prehabilitation protocols, fostering a more proactive and personalized approach to surgical planning. Such prehabilitation could help significantly decrease IBR failure, especially in prophylactic mastectomy [66–68].

Despite the strengths of our approach, several limitations should be acknowledged. First, the model was developed using retrospective single-center data, which may limit its generalizability across diverse populations or institutions with different protocols and surgical expertise. Second, although rigorous internal validation was performed, external validation is essential before clinical implementation at scale. Large prospective international multicenter cohorts in the future would further endorse the use of this predictive score. The inclusion period was prolonged (2006–2023) which could have exposed it to temporal biases. However, the year of surgery was analyzed as a covariate to rule out major biases. Another major limit of this work is postoperative radiation. Our model was based on a retrospective cohort. Therefore, all cases of postoperative radiotherapy were recorded. In the case of a preoperative score calculation, this variable is not always known. Despite greater predictability during preoperative multidisciplinary discussions and advanced imaging, some cases will require decision of postoperative radiation therapy only following final histopathology. We suggest calculating two scores (with and without postop radiation) in cases where planned adjuvant radiotherapy is unsure, to implement the preoperative discussion with relevant numbers. Finally, implementing more relevant variables, such as intraoperative perfusion assessment of the skin flap, surgeon experience, or patient-reported outcomes, could enhance the overall relevance. Although the type of mastectomy and skin flap management was not included in the final predictive equation, this decision reflects a data-driven modeling approach where variable selection was based on statistical performance and redundancy minimization. Likely, the predictive information load associated with this variable was already captured by other selected variables, thereby limiting its marginal utility in the final model. The following key step will be integrating the predictive score into clinical practice, either through developing a dedicated digital application or incorporating it into electronic health record systems. Such tools could enable automatic risk calculation at the point of care, supporting real-time surgical planning and patient counseling. Prospective multicenter validation is needed to confirm generalizability, but implementing this score into clinical workflows could promote more consistent, evidence-based decision-making. In the future, integrating clinical, radiological, and patient-reported data may further enhance the model’s predictive power and broaden its applicability across different reconstructive pathways.

## Conclusion

We developed and internally validated a machine learning-based model that provides an accurate prediction of IBR failure within 12 months. By leveraging elastic net regularization and adhering to TRIPOD + AI reporting standards, the model demonstrated strong discrimination and calibration. Its integration into a preliminary online calculator will help real-time individualized risk estimation, potentially enhancing preoperative planning, fair information, prehabilitation, and shared decision-making. This strong basis suggests prospective external validation and implementation studies, warranted to confirm its clinical utility and impact on outcomes.

## Supplementary Information

Below is the link to the electronic supplementary material.

Supplementary file1 (PNG 6 kb)

Supplementary file2 (DOCX 15 kb)

Supplementary file3 (DOCX 15 kb)

Supplementary file3 (DOCX 15 kb)

## Abbreviations

AI: Artificial intelligence
AUC: Area under the curve
BMI: Body mass index
BR: Breast reconstruction
IBR: Implant-based breast reconstruction
ROC: Receiver operating characteristics
