# Supplementary figures and images for: Machine Learning to Predict Implant-Based Breast Reconstruction Failure: A Bootstrap-Validated Elastic Net Model
Source: Aesthetic Plast Surg. 2026 Apr 13;50(11):4097–110. doi: 10.1007/s00266-026-05795-2 (PMC13315410; doi:10.1007/s00266-026-05795-2)

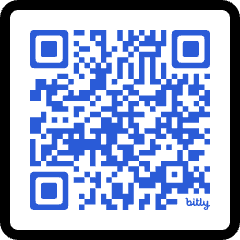

Supplement: Supplementary file 1 — Supplementary file1 (PNG 6 kb) [file 266_2026_5795_MOESM1_ESM.png]
