# Supplementary material for: Machine Learning to Predict Implant-Based Breast Reconstruction Failure: A Bootstrap-Validated Elastic Net Model
Source: Aesthetic Plast Surg. 2026 Apr 13;50(11):4097–110. doi: 10.1007/s00266-026-05795-2 (PMC13315410; doi:10.1007/s00266-026-05795-2)
Supplement: Supplementary file 2 — Supplementary file2 (DOCX 15 kb) [file 266_2026_5795_MOESM2_ESM.docx]

**Supplementary Table 1. Acellular dermal matrices and meshes used in implant-based breast
 reconstruction in this study.**

| Code | Brand | Model | Country | Type | Origin |
| --- | --- | --- | --- | --- | --- |
| 1 | Decomed | Braxon | Italy | ADM | Bovine dermis |
| 2 | Surgimend | PRS | USA | ADM | Fetal bovine dermis |
| 3 | Surgimend | PRS Meshed | USA | ADM | Fetal bovine dermis |
| 4 | Synovis | Veritas | USA | Collagen Matrix | Bovine Pericardium |
| 5 | Assut Europe | Bioriopar | Italy | ADM | Porcine dermis |
| 6 | Allergan | Strattice RTM | USA | ADM | Porcine dermis |
| 7 | Tecnoss | Protexa | Italy | ADM | Porcine dermis |
| 8 | DecoMed | Native | Italy | ADM | Porcine dermis |

*ADM : Acellular Dermal Matrix*
