# Supplementary material for: Machine Learning to Predict Implant-Based Breast Reconstruction Failure: A Bootstrap-Validated Elastic Net Model
Source: Aesthetic Plast Surg. 2026 Apr 13;50(11):4097–110. doi: 10.1007/s00266-026-05795-2 (PMC13315410; doi:10.1007/s00266-026-05795-2)
Supplement: Supplementary file 3 — Supplementary file3 (DOCX 15 kb) [file 266_2026_5795_MOESM3_ESM.docx]

**Supplementary Table 2. Sample size calculation.**

| Sample Size Estimation for Model Development*^1^* | | | | | |
| --- | --- | --- | --- | --- | --- |
| Criteria | Sample Size | Shrinkage | CS R-squared | Nagelkerke R-squared | Events per Predictor Parameter (EPP) |
| Criterion 1 | 411 | 0.850 | 0.1505 | 0.264 | 5.140 |
| Criterion 2 | 382 | 0.841 | 0.1505 | 0.264 | 4.770 |
| Criterion 3 | 196 | 0.850 | 0.1505 | 0.264 | 2.450 |
| Final | 411 | 0.850 | 0.1505 | 0.264 | 5.140 |
| *^1^* Minimum sample size required for new model development is 411 with 62 events (given input C-statistic = 0.8 and assuming prevalence = 0.15 with 12 candidate parameters), and an EPP=5.14.  Given input C-statistic = 0.8 & prevalence = 0.15 ; Cox-Snell R-sq = 0.1505  *NB: Assuming 0.05 acceptable difference in apparent & adjusted R-squared*  *NB: Assuming 0.05 margin of error in estimation of intercept*  *NB: Events per Predictor Parameter (EPP) assumes prevalence = 0.15* | | | | | |
