# Supplementary material for: Machine Learning to Predict Implant-Based Breast Reconstruction Failure: A Bootstrap-Validated Elastic Net Model
Source: Aesthetic Plast Surg. 2026 Apr 13;50(11):4097–110. doi: 10.1007/s00266-026-05795-2 (PMC13315410; doi:10.1007/s00266-026-05795-2)
Supplement: Supplementary file 4 — Supplementary file3 (DOCX 15 kb) [file 266_2026_5795_MOESM4_ESM.docx]

| **Model** | **Optimism Corrected AUC** | **Calibration Curve** |
| --- | --- | --- |
| Elastic Net Logit Model | 0.8117814 | Very Good |
| Elastic Net Probit Model | 0.8077076 | Good |
| Elastic Net Cauchit Model | 0.7814259 | Very Poor |
| Elastic Net Gompit Model | 0.7889442 | Very Poor |
| Lasso Logit Model | 0.8051151 | Very Good |
| Ridge Logit Model | 0.7993993 | Poor |
| Standard Logit Model | 0.8049059 | Good |
| Stepwise Logit Model | 0.7644526 | Poor |
| Random Forest Model | 0.9417283 | Very Poor |
| Support Vector Machine Model | 0.8555071 | Very Poor |
| k-Nearest Neighbors Model | 0.8564404 | Poor |
| Linear Discriminant Analysis | 0.7900163 | Poor |
| Logistic Regression on PCA | 0.8230584 | Good |

**Supplementary Table 3. Summary table for all the optimism-corrected AUCs.** Multiple models were developed and evaluated by bootstrap validation. The choice of the final model presented also takes into account aspects from Decision Curve Analysis theory, and the ease of implementation in clinical practice permitted by a simple equation rendered by the Elastic Net calculated coefficients
